# A High Resolution Case Study of a Patient with Recurrent *Plasmodium vivax* Infections Shows That Relapses Were Caused by Meiotic Siblings

**DOI:** 10.1371/journal.pntd.0002882

**Published:** 2014-06-05

**Authors:** Andrew Taylor Bright, Micah J. Manary, Ryan Tewhey, Eliana M. Arango, Tina Wang, Nicholas J. Schork, Stephanie K. Yanow, Elizabeth A. Winzeler

**Affiliations:** 1 Biomedical Sciences Program, School of Medicine, University of California, San Diego, La Jolla, California, United States of America; 2 Department of Pediatrics, School of Medicine, University of California, San Diego, La Jolla, California, United States of America; 3 Scripps Genomic Medicine, The Scripps Translational Science Institute, La Jolla, California, United States of America; 4 Department of Molecular and Experimental Medicine, The Scripps Research Institute, La Jolla, California, United States of America; 5 Grupo Salud y Comunidad, Facultad de Medicina, Universidad de Antioquia, Medellín, Antioquia, Colombia; 6 School of Public Health, University of Alberta, Edmonton, Alberta, Canada; 7 Provincial Laboratory for Public Health, Edmonton, Alberta, Canada; Federal University of São Paulo, Brazil

## Abstract

*Plasmodium vivax* infects a hundred million people annually and endangers 40% of the world's population. Unlike *Plasmodium falciparum*, *P. vivax* parasites can persist as a dormant stage in the liver, known as the hypnozoite, and these dormant forms can cause malaria relapses months or years after the initial mosquito bite. Here we analyze whole genome sequencing data from parasites in the blood of a patient who experienced consecutive *P. vivax* relapses over 33 months in a non-endemic country. By analyzing patterns of identity, read coverage, and the presence or absence of minor alleles in the initial polyclonal and subsequent monoclonal infections, we show that the parasites in the three infections are likely meiotic siblings. We infer that these siblings are descended from a single tetrad-like form that developed in the infecting mosquito midgut shortly after fertilization. In this natural cross we find the recombination rate for *P. vivax* to be 10 kb per centimorgan and we further observe areas of disequilibrium surrounding major drug resistance genes. Our data provide new strategies for studying multiclonal infections, which are common in all types of infectious diseases, and for distinguishing *P. vivax* relapses from reinfections in malaria endemic regions. This work provides a theoretical foundation for studies that aim to determine if new or existing drugs can provide a radical cure of *P. vivax* malaria.

## Author Summary

*Plasmodium vivax* is capable of remaining dormant in the human liver for months to years after an initial infection, creating an asymptomatic human reservoir. This unique aspect of parasite biology makes eliminating *P. vivax* distinctly different from *P. falciparum* elimination, and yet very little is known about this dormant parasite stage. Lack of knowledge about the dormant liver stage prevents the creation of new drugs and public health interventions directed at *P. vivax*. In order to better understand this particular parasite stage, we used whole genome sequencing to analyze three sequential *P. vivax* infections, two of which could be definitively categorized as having arisen from dormant liver stages. Our whole genome sequencing data demonstrates that dormant liver stage parasites are closely related yet not, as had previously been postulated, identical. These data highlight the need for a new paradigm to investigate *P. vivax* dormant liver stages in order to design the next generation of *P. vivax* drugs and effective global health interventions.

## Introduction

*Plasmodium vivax* is the most widespread of the human malaria parasite species with 2.85 billion people living in areas at risk for *P. vivax* infection [1]. Worldwide there are approximately 100 million cases of *P. vivax* annually, and the severity of *P. vivax* infection is increasingly recognized as more cases of death and drug resistance are reported [2]. The predominant biological mechanism that accounts for the increased range of *P. vivax* is the ability of these parasites to persist as dormant liver stages known as hypnozoites. This unique parasite stage is metabolically inactive and can remain dormant for months to years before reemerging to cause clinical disease [3], [4]. Asymptomatic hypnozoite carriers therefore represent a major impediment to malaria elimination efforts.

Despite the large burden of *P. vivax* malaria throughout the world, little is definitively known about hypnozoite reactivation. Recent reports in the literature have shown that relapses occurring in patients with a low number of hypnozoites in their liver are usually clonal and the relapse parasites are genetically homologous to the parasites from the initial infection [5]–[7]. In contrast most relapse infections in endemic settings, where patients harbor hypnozoites from multiple infectious mosquito bites, are polyclonal infections caused by parasites genetically heterologous to the initial infection [8]–[10]. The heterologous infections do share some alleles suggesting the parasites share a common ancestor [8] but the polyclonal nature and higher allelic diversity [11] of these infections along with the limited number of genetic markers used in previous studies make it difficult to assess the specific genetic relationship.

The complex dynamics of *P. vivax* relapse infections prevents using genotyping methods to classify recurrent malaria episodes in the field as relapse infections caused by hypnozoites, as recrudescent infections caused by a failure to clear the initial infection, or as reinfections. The inability to distinguish between the three causes of recurrent malaria infection in endemic areas prevents accurate estimates of hypnozoite prevalence and inhibits the ability to study this parasite stage directly. Furthermore, the inability to distinguish relapse infections from reinfections prevents drug efficacy trials in endemic countries, impeding the development of the next generation of anti-hypnozoite drugs and hindering a thorough understanding of resistance to primaquine, the only currently licensed drug able to clear hypnozoites and achieve a radical cure.

Due to these confounding aspects of relapse infection, studies of travelers who move into an endemic region, contract malaria once, and then leave could be particularly informative. Here we report the analysis of whole genome sequencing data from sequential recurrent parasite infections obtained from a patient who had a *P. vivax* malaria episode shortly after arriving in Canada from Sudan (where the infection occurred), and subsequently experienced two relapses, 3 months and 33 months after the first episode, despite treatment with the recommended drug regimen. Analysis of single nucleotide variants (SNVs) identified in the three recurrent malaria infections demonstrate that while the first recurrent infection was polyclonal, the two subsequent infections, which can be definitively categorized as relapse infections, were clonal. In addition, comparison of the SNVs identified in the three infections demonstrate that the parasites isolated from the patient are most likely meiotic siblings and are the result of a single sexual cross in the mosquito vector.

## Materials and Methods

### Ethics statement

The protocol used to collect human blood samples for this work was approved by the Health Research Ethics Board of the University of Alberta and written informed consent was obtained from the patient. The consent form states in English that blood samples collected from the patient may be used to genetically characterize the parasites and that samples may be shared with other researchers for scientific purposes.

### Sample collection

*P. vivax* DNA used in this study was isolated from a symptomatic patient who was blood smear positive for *P. vivax* malaria in Alberta, Canada as previously described [12]. The patient was a 38-year-old male originally from Eritrea. In December 2008 he spent nearly one month in Sudan where, according to patient history, he contracted malaria for the first time in his life. The patient was treated with chloroquine but not primaquine. After treatment, he relocated to Canada in mid-January 2009 and experienced his first recurrent episode (EAC01) within two weeks of arrival. He was treated with chloroquine (600 mg base immediately, 300 mg base at 6, 24, and 48 h) and primaquine (30 mg daily P.O. for 14 d) as standard clinical care. The patient recovered clinically and was blood smear negative for malaria on day 16. Subsequently, the patient experienced two relapse episodes within 3 months (EAC02) and 33 months (EAC03) of his initial recurrent infection in Canada. During EAC02, the patient was treated with the same regimen of chloroquine as before but was given an extended 28 d dose of primaquine (30 mg P.O. daily) and was blood smear negative after two days. During the final episode of this study, the patient was treated with chloroquine for three days and received standard primaquine (14 d 30 mg P.O. daily) and was again blood smear negative after two days and recovered clinically. At each episode, EDTA-preserved blood was collected from the patient and submitted to the Provincial Laboratory for Public Health (Edmonton, Canada) for routine confirmation of malaria by real-time PCR. Blood from the first two episodes was stored at −20°C prior to use for sequencing. Whole blood from the third episode was centrifuged to pellet red blood cells and stored at −80°C.

### Genotyping of eight genetic makers

Parasite DNA was extracted from 40 µL of whole blood using the PSS GC12 instrument (Precision System Science Co. Ltd.) with the DNA 200 protocol and kits (E2003). DNA was eluted into a 100 µL volume. Genotyping was based on sequence repeats in the microsatellites 1.501, 3.502, 3.27, and MS16, as well as the genes msp1F3, msp3α, msp4 and msp5 using the primers and protocol as previously described [13]. All molecular markers were amplified by nested or semi-nested PCR using 3 µL of extracted DNA as a template in the first amplification step and 1 µL of the first PCR product for the second amplification. The PCR reaction was performed in a final volume of 20 µL containing 1X PCR buffer (Qiagen), 2 mM of MgCl2 (Qiagen), 200 µM of each dNTP (Takara Bio), 0.25 µM of each primer, and 1.5 units of HotStar Taq DNA polymerase (Qiagen). The cycling program was as follows: 5 min at 95°C followed by 30 cycles of denaturation for 1 min at 95°C, annealing for 1 min at 56°C–62°C (depending on the marker analyzed), and elongation for 1 min at 72°C with a final elongation for 5 min at 72°C.

PCR was performed in a Thermal Cycler 2720 (Applied Biosystems). Amplification was confirmed in a 2% agarose gel and PCR products were stored at 4°C in the dark. The product size was resolved by capillary electrophoresis in an ABI Prism 3100 Genetic Analyzer (Perkin Elmer Applied Biosystems), using GS500 LIZ as the internal size standard and the microsatellite settings. The results were analyzed using GeneMapper software (version 3.5; Applied Biosystems). All electropherograms were inspected visually and peaks above a cut off of 300 relative fluorescent units (RFU) were considered true amplification products. Based on the repeat length, alleles were grouped into 3-bp bins for MS16, msp1F3, msp3α, msp4 and msp5, 4-bp bins for 3.27, 7-bp bins for 1.501 or 8-bp bins for 3.502. Multiple alleles per locus were scored if minor peaks were >33% of the height of the predominant allele present for each locus.

### Isolation and quantification of genomic DNA for whole genome sequencing

For samples EAC01, EAC02, and EAC03, bulk genomic DNA was isolated from frozen whole blood samples using the DNeasy Blood and Tissue kit (Qiagen) as per the manufacturers instructions.

A Taqman qPCR assay for *P. vivax* b-tubulin (PVX_094635) was used to assess the *P. vivax* DNA quantity in the bulk gDNA isolated from the patient blood sample (Primer 1: CGAAAGGAAGCAGAAGGATG and Primer 2: GGGGAGGGGAATACTGAAAA with a Hydrolysis Probe of CAGGTAGTGGTATGGGAACCTTGCTGA). The qPCR reaction was conducted using Applied Biosystems Taqman 2× Genotyping Master Mix (Life Technologies), 20 ng bulk genomic DNA, 900 nM of each primer, and 250 nm of the fluorescent hydrolysis probe. Reactions were carried out on an Applied Biosystems StepOne Plus (Life Technologies) using the manufacturers standard protocol. A 12-point standard curve was made from Sal1 reference DNA originally obtained from the CDC by serially diluting 20 ng of Sal1 gDNA 1∶2 for a theoretical lower limit of quantitation of 0.02% *P.vivax* DNA. Total *P vivax* DNA was calculated by comparing the Ct value of the sample to the 12-point standard curve of Sal1 reference DNA.

### Whole genome capture

Whole genome capture (WGC) of the initial infection and the relapse samples was performed as previously described [14]. Briefly, Illumina TruSeq v. 3-style Y-adaptors (CCACTCATGCAGGTGAGCGTC*T and /Phos/GACGCTCACCTATGTCTCCCT) were ligated onto Sal1 reference genomic DNA that had been sheared to 200 bp using an S-series Covaris Adaptive Focused Acoustic machine (Covaris). The T7 promoter sequence (bold) was added into the standard Illumina amplification primers (TTC[**TAATACGACTCACTATAGGG**]AGACATAGGTGAGCCTC and CCACTCATGCAGGTGAGCGTCT) used to amplify the ligated products. To create the whole genome baits, the resulting library was used in an in vitro transcription reaction following the manufacturers protocol (Ambion MEGAshortscript T7 Kit, Life Technologies) with the exception that biotin labeled dUTP was used in replacement of the supplied dUTP.

Bulk genomic DNA was carried through the standard Illumina whole genome sequencing (WGS) library preparation process using Adaptive Focused Acoustics for shearing (Covaris), end-repair, A-tailing and ligation (New England Biolabs). Hybridization capture was carried out as previously described [14], [15]. Briefly, 750 ng of the whole genome baits were incubated with 500 ng of the bulk genomic DNA-fragment library along with 2.5 µg of human Cot-1 DNA, 2.5 µg of salmon sperm DNA, 2.5 ug of Human genomic DNA, and 1 unit of blocking oligonucleotides complementary to the Illumina TruSeq v. 3 adaptor and incubated for 24 hours at 65°C. After the hybridization, the captured targets were selected by pulling down the biotinylated probe/target hybrids by using streptavidin-coated magnetic beads (Dynabeads MyOne Streptavidin T1; Life Technologies) as previously described [14].

### Whole genome sequencing and data analysis

Whole genome capture samples were sequenced on an Illumina Hi-Seq2000 at the TSRI Next Generation Sequencing Core Facility. Samples were paired-end sequenced for 101 bp per read and one 7 bp index read using Illumina v. 3 chemistry. Base calls were made using Illumina RTA (v. 1.12) software. Data for each sample sequenced in this study is available in the NCBI Sequence Read Archive [SRA057904].

Fastq files obtained from sequencing were aligned to the Sal1 reference using BWA (v. 0.5.9) with soft clipping of bases with quality score 2 and below [16]. PCR duplicates were next identified and marked using Picard (v. 1.51) MarkDuplicates. Aligned reads were then realigned around indels and areas of high entropy using GATK (v. 1.3+) IndelRealigner, and the base quality scores of realigned reads were then recalibrated using GATK TableRecalibration [17], [18]. After realignment and recalibration the samples were considered “clean” and ready for use in downstream analysis.

Genome wide coverage and loci covered to a certain percentage were calculated using GATK DepthOfCoverage [18]. For all GATK DepthOfCoverage analyses the minimum mapping quality (mmq) was set to 29 and the minimum base quality (mbq) was set to 20.

SNV discovery was conducted on the ten publicly available *P. vivax* genomes [North Korea I: SRP000316, Mauritania I: SRP000493, Brazil I: SRP007883, India VII: SRP007923, IQ07: SRP003406, SA94–SA98: SRA047163]. Only those reads from each sample that aligned in proper pairs were used in the SNV discovery process (samtools view –f 2) [19]. SNVs were identified in each sample individually using GATK UnifiedGenotyper and stringent filters were applied to achieve the highest confidence SNV set possible with GATK VariantFiltration. The filters used included minimum depth of coverage of 20, minimum ReadDepthAndAllelicFractionBySample of 1.0, maximum Fisher's Exact test for strand bias of 3.0, and maximum HaplotypeScore of 3.0. Additionally SNVs were required to be biallelic with respect to Sal I and SNVs that were within 50 bp of each other were both excluded. The resulting 10 high stringency genotype sets were combined into a single set of 55,399 high confidence SNVs.

The 55,399 high confidence SNVs identified in the SNV discovery process outlined above were then genotyped in all three samples using GATK UnifiedGenotyper. Those loci with multi-allelic genotypes were used only for analysis of clonality. The resulting VCF file was annotated using SnpEff v. 3.3 (snpeff.sourceforge.net) [20] and principal components analysis and all SNV plots were completed with MATLAB v. 7.12.0.635 (The Mathworks).

For the F_ws_ calculation the reference and alternate read depths were extracted from the VCF file and used in Equation 1 where p_w_ and p_s_ are the allelic frequency of the reference allele within the sample and within the population, respectively, and q_w_ and q_s_ are the allelic frequency of the alternate allele within the sample and within the population, respectively [21].
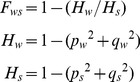
(1)


Regions of contiguous DNA that were identical between samples were identified and a weighted average block size metric (HapBlockMet) was calculated for each pairwise comparison using all identified haplotype blocks according to Equation 2 where *d* is the length of the block.
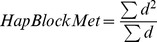
(2)


Copy number variants were detected using a novel CNV detection algorithm [22]. Briefly, after reads were aligned to the reference genome, depth of coverage was normalized for GC bias across the entire genome excluding the apicoplast and mitochondria. Regions were considered amplified if the average of continuous bases normalized by a Gaussian curve with standard deviation of 50 bases showed a two fold or greater read coverage relative to the rest of the genome. Genome fold coverages were analyzed in a per region fashion, with each region that had a statistically significantly higher coverage (p<0.05, normalized for number of regions, two-proportion z-test compared to average) being called as a copy number variant. The size of the region was varied, with the first and last base pair positions being considered the boundaries of the CNV, and the region that produced the most significant result was considered to have the true CNV boundaries.

## Results

### Analysis of sequential recurrent *P. vivax* infections

The patient, a 38-year-old male from Northeast Africa, moved to Canada in mid-January 2009 and presented with *P. vivax* malaria one month after experiencing his first primary infection in Sudan. During this initial recurrent infection, the patient was treated with the recommended regimen of both chloroquine and primaquine. After subsequent recovery, the patient presented with *P. vivax* malaria again three months later and was treated with chloroquine and an extended 28-day course of primaquine. Thirty months later the patient presented with a third recurrent *P. vivax* malaria infection and was given a standard dose of chloroquine and primaquine. Throughout the three malaria infections the patient had not travelled outside of areas in North America known to be non-endemic for malaria, thereby ruling out reinfection [12].

According to the patient's medical history, the first recurrent infection (EAC01) was classified as either a recrudescence caused by a failure to clear parasites from the primary infection in Sudan or a relapse infection caused by reactivation of a dormant hypnozoite. The second (EAC02) and third (EAC03) recurrent infections were classified as relapse infections since parasites were cleared from the patient after each preceding infection in Canada. Infected blood samples were collected during each malaria episode and frozen. Because of the unique history of this patient, the samples were thawed and examined after the third malaria episode.

### Whole genome sequencing

Although the *P. vivax*-infected patient blood samples had not been collected using the leukocyte depletion protocol required for efficient direct sequencing analysis of *P. vivax* samples [23], [24], we were able to sequence the three parasite strains using a whole genome capture technique utilizing RNA baits derived from the SalI reference strain of *P. vivax* [14] using an in-solution hybridization capture procedure [15], [25]. Briefly, in this method the *P. vivax* DNA from a patient sample hybridizes to the biotinylated RNA baits and the DNA/RNA hybrids are then purified using streptavidin beads, resulting in the depletion of most of the contaminating human DNA. This method enriched the *P. vivax* DNA, which initially comprised less than 1.0% of the total genomic DNA (gDNA) in the patient infected whole blood samples, to 20%–40% of total DNA content allowing efficient whole genome sequencing analysis of the parasite DNA (Table 1). Enriched *P. vivax* gDNA was sequenced on an Illumina HiSeq 2000 using 100 base-pair paired end reads and 5.1–8.5 billion bases were obtained for each parasite strain resulting in genome wide coverage of 35X–118X (Table 1). In addition, for all three samples, >88% of the genome could be assigned a confident genotype (Table 1).

**Table 1:** Sequencing statistics for whole-genome capture samples.

Isolate	Sequenced bases (billion)	*P. vivax* gDNA (%)	PCR duplicates (%)	Coverage (X)	Genome Callable^a^ (%)
EAC01	5.1	19.57%	14.20%	34.81	88.2%
EAC02	6.0	36.48%	12.41%	75.96	94.0%
EAC03	8.5	39.67%	11.89%	118.22	96.0%

a Base pair covered by 5 or more reads.

**Figure 1:**
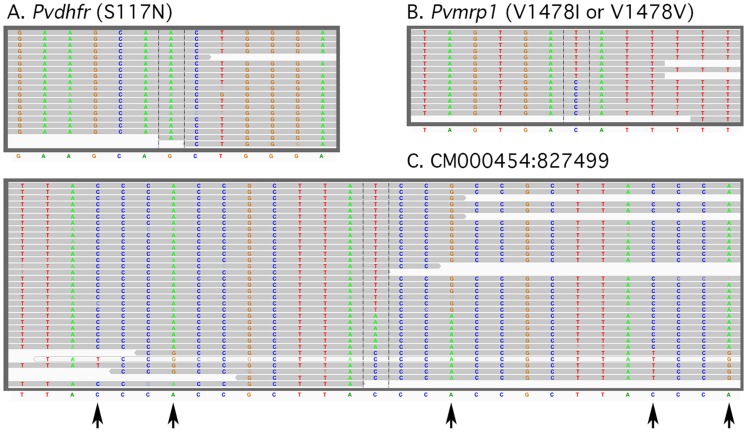
Representative genotype calls. Examples of read alignments from (A) a homozygous SNV (S117N) in *P. vivax dihydrofolate reductase* (PVX_089950), (B) a heterozygous SNV in the *P. vivax multidrug resistance protein 1* (PVX_097025) and (C) a rare multiallelic SNV on chromosome 13 that is suggestive of an alignment error based on multiple mismatches (indicated by arrows) in the fragments carrying the G at the central position. All reads are from EAC01. The Sal I reference allele is shown below.

Several types of reads were evident in comparison to the SalI reference sequence. These include clear homozygous single nucleotide variants such as the one causing the S117N change in the *P. vivax dihydrofolate reductase* gene (PVX_089950) (Figure 1A), biallelic reads (Figure 1B), such as the “T” in the isoleucine codon and the “C” in the valine codon at amino acid 1478 in the *P. vivax multidrug resistance associated protein* (PVX_097025), and multiallelic reads in a noncoding region on chromosome 13 (Figure 1C). Although the biallelic reads appeared real and, in many cases, involve alleles present in other *P. vivax* isolates, multiallelic reads, such as that shown in Figure 1C, appeared to be due to alignment errors based on the fact that numerous mismatches are found throughout the read. These alignment errors were subsequently removed by excluding cases where there were more than 1 SNV in 50 bases. These misalignments were not used in further analyses. The single and biallelic reads, their readcount, and their position in the genome are given in Supporting Dataset 1.

### Genotyping of single nucleotide variants

In order to examine the origins of the parasites, we first sought to identify the genetic differences between the three infections by examining their genome sequence. Using the whole genome sequencing data, we genotyped the three *P. vivax* infections at 55,399 positions (Table 2). These loci were selected by analyzing the genome sequences of 10 diverse *P. vivax* strains from the NCBI Sequence Read Archive and identifying the location of SNVs that differed from the SalI reference sequence. SNVs included in this set of markers were required in at least one of the 10 sequences to have a minimum coverage of 20 reads and all reads indicating the presence of a single allele. These markers were spaced, on average, 408 bases apart across the genome and the distribution was similar across all chromosomes (data not shown). Approximately 44% of the SNVs genotyped were located in coding regions, which constitute 54.6% of the genome (Table 2).

**Table 2:** SNV genotyping set: East African sample SNV statistics.

	EAC01	EAC02	EAC03
Genotyped loci	55,399	55,399	55,399
Total SNVs	23,379	20,734	20,934
SNVs covered >5 reads	19,667	19,623	20,110
Homozygous SNVs	11,064	19,365	19,807
Heterozygous SNVs	8,603	258	303
% Heterozygous	43.74%	1.31%	1.51%
F_ws_	0.51	0.97	0.94
% Heterozygous SNVs in variable regions	15.65%	35.37%	41.45%
% Genome consisting of variable regions	11.97%	11.97%	11.97%
# SNVs in coding regions^a^	10,401	9,102	9,147
# Synonymous SNVs^a^	4,248	3,674	3,682
# Non-synonymous SNVs^a^	6,153	5,428	5,465
Base pairs per SNV^a^	967	1,091	1,080
% SNVs in coding regions^a^	44.5%	43.9%	43.7%
% of genome consisting of coding regions^b^	54.6%	54.6%	54.6%

a Only homozygous SNVs are used in the calculation.

b Genome size - 22,621,101 bp; Coding regions - 12,348,368 bp.

The samples analyzed here were different from the SalI reference genome at 23,379, 20,734, and 20,934 of the genotyped loci for EAC01, EAC02, and EAC03, respectively (Table 2). Of these variant loci, 19,667, 19,623, and 20,110 had five or more reads mapping to the locus in EAC01, EAC02, and EAC03, respectively, and were considered high quality genotype calls (Table 2).

**Figure 2:**
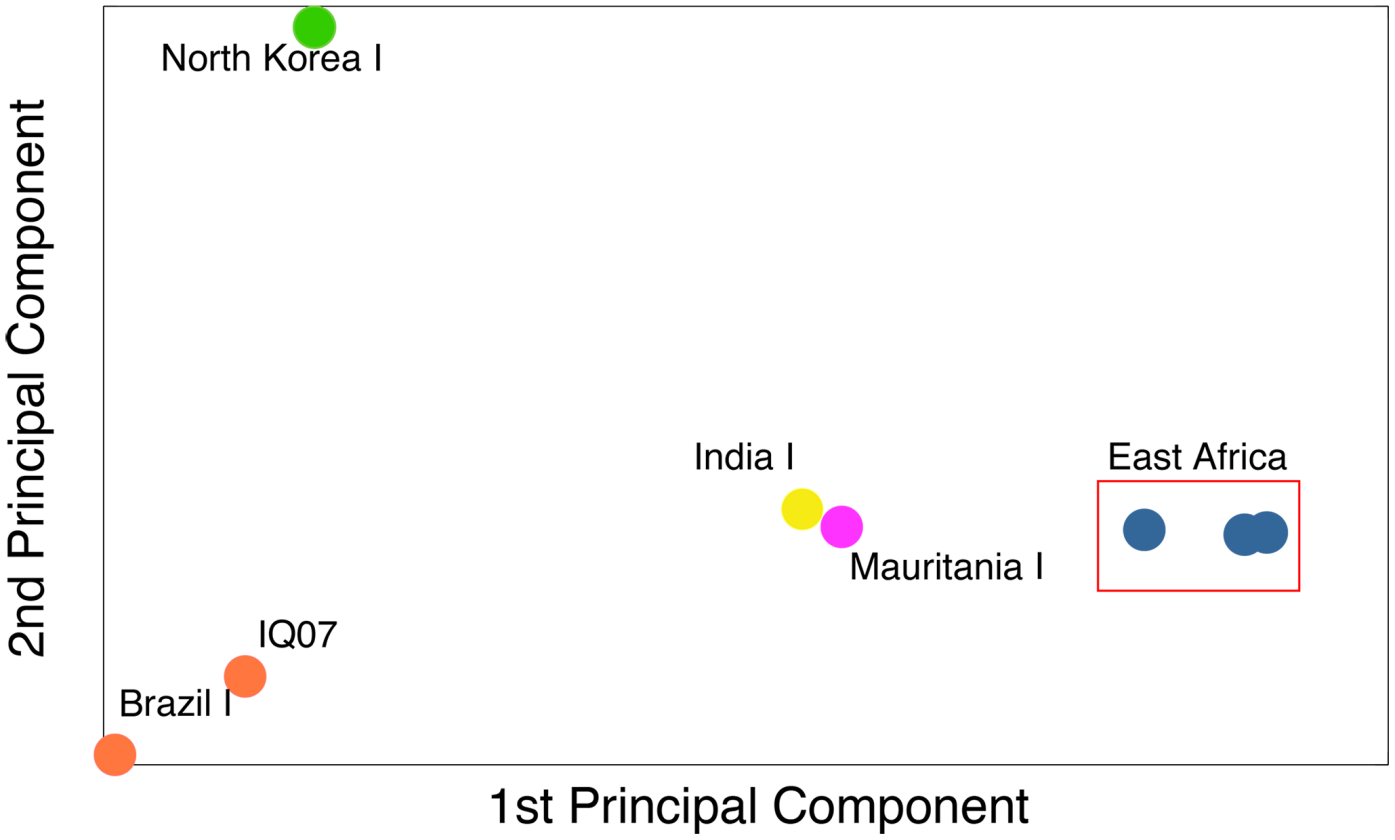
Principal components analysis. The East African samples sequenced in this study cluster by themselves as compared to the publicly available *P. vivax* samples that possess geographic information [North Korea I: SRP000316, Mauritania I: SRP000493, Brazil I: SRP007883, India VII: SRP007923, IQ07: SRP003406]. They are most closely related to IndiaVII and Mauritania I (West Africa). They are highly diverged from North Korea I and the South American strains. Principal components analysis was performed using MATLAB.

In order to establish that all the infections were of East African origin, as suggested by the patient's medical history, we compared the three infections isolated here with five geographically diverse *P. vivax* strains for which both sequencing data and geographical data are publicly available using the same 55,399 loci [23], [26]. Using principal components analysis [27], we show that our three parasite strains from East Africa are very closely related to one another (Figure 2). In addition, the three samples from our patient are most closely related to the West African Mauritania I and India VII strains and are diverged from *P. vivax* samples from South America and the North Korea I strain (Figure 2). Principal components analysis corroborates the patient's medical history and offers further proof that he was not reinfected by an unrelated *P. vivax* strain while residing in Canada.

### Analysis of SNVs: Clonality

With the dense set of genetic markers obtained from WGS data we next sought to establish the clonality of the three infections. For this analysis we used both single and biallelic genotype calls in EAC01, -02, and -03, and calculated the percentage of loci containing more than one allele in this haploid organism as an indicator of clonality. The parasites in the first blood sample (EAC01) were determined to be polyclonal with 43.7% (8,603 of the 19,667 variant loci with 5 or more reads) possessing more than one allele (Table 2). In addition, the F_ws_ value, another indicator of multi-clonal infections which compares the parasite diversity within a single patient to the parasite diversity seen on the population level [21], [28], is 0.51 (clonal = 1.0) for the initial infection and is suggestive of more than one clone (Table 2) [28].

In contrast, both relapses arising from hypnozoites activated 30 months apart (EAC02 and EAC03) appeared to be clonal based upon the same metrics as above (Table 2), which is consistent with previous reports of relapse infections in non-endemic settings [6], [29]. The first relapse (EAC02) had an F_ws_ of 0.97 and the second relapse had an F_ws_ of 0.94 (Table 2). In addition both relapse samples had very few loci with multiple alleles (258 (1.31% of total SNVs) for EAC02, 303 (1.51% of total SNVs) for EAC03) at the variant genotyped loci (Table 2). For EAC03, 69% (45) of the 303 multi-allelic loci also gave mixed reads in one of the other sequenced samples (e.g. India VII, IQ07, Brazil 1, North Korea or Mauritania) suggesting these loci are in an area of the *P. vivax* genome which is problematic to sequence using current short-read technology. In addition, more than 35% of these multi-allelic loci in EAC02 and EAC03 were clustered in subtelomeric regions and regions encoding internal variable gene families, which comprise only 12% of the *P. vivax* genome (Table 2). Also of note, the distribution of multi-allelic loci was nonrandom with many of the mixed read alleles mapping to one 70 kb fragment on the right arm of chromosome 7, suggesting that this region might be duplicated in the three East African isolates. We therefore conclude that these few loci containing multiple alleles in the clonal samples are most likely the result of sequencing/alignment errors to these highly variable sequences, which frequently duplicate and recombine during mitotic growth [30].

**Figure 3:**
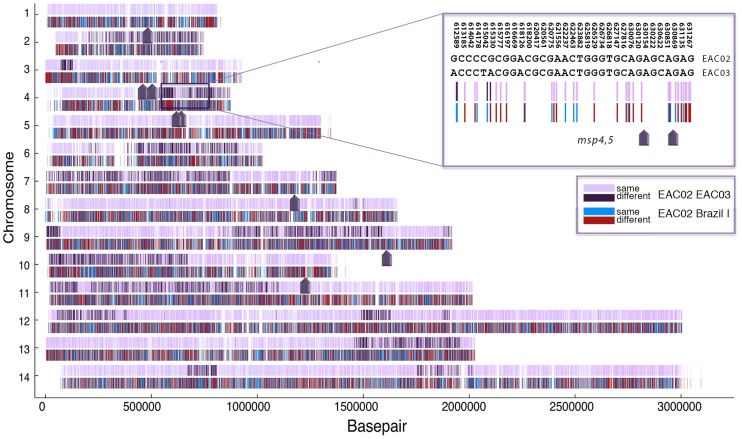
Pairwise comparison of EAC02 and EAC03. A set of 23,755 SNVs confidently genotyped (20 or more reads) in EAC02, EAC03 and Brazil I and which are different from SalI are presented across 14 chromosomes. SNVs shared between the two strains are shown in lavender and positions where the two strains differ are shown in dark purple, revealing large regions of identity. The positions of the eight-microsatellite markers (Table 3) are indicated by arrows, including the region on chromosome 4 where the *msp4* and *msp5* markers are in a region of homozygosity surrounded by regions of heterozygosity (inset). Despite the two samples being identical at the eight microsatellite markers, they differ at many positions in the genome. A comparison between EAC02 and a Brazil I control strain (in red and blue) shows that the two strains are highly diverged and do not share blocks of contiguous DNA sequence.

To compare these findings using conventional methods, the three *P. vivax* infections were subjected to eight-marker RFLP genotyping, which represents the current standard in characterizing *P. vivax* diversity. The markers used here consisted of four microsatellites and four genes of the highly variable *merozoite surface protein* family, all of which have been shown to be variable in previous studies [13], [31], [32]. These regions were amplified using PCR (see methods), and the size of the PCR products were analyzed on an ABI Prism 3100 Genetic Analyzer. As expected, the first infection (EAC01) showed multiple bands for 6 of the 8 markers, while EAC02 and EAC03 appeared monoclonal, with only a single band for each of the 8 markers for EAC02, and 7 of the 8 markers for EAC03, where three bands were observed for the 3.27 microsatellite marker (Table 3). It seems unlikely that this extra band is informative given that the regions appear perfectly identical in EAC02 and EAC03 in this region by whole genome sequencing (Figure 3). It is possible that the extra bands for the 3.27 marker are due to either contamination or PCR artifacts resulting from mis-hybridization of the PCR primers for this marker in the East African isolates. These data suggest that microsatellite genotyping of field isolates, although standard, may lead to inaccurate conclusions about polyclonal infections.

**Table 3:** Genomic location and band size of eight genetic markers in *P. vivax* [37].

		Primer Location (bp)				
Genetic Marker	Chrom.	Forward	Reverse	EAC01	EAC02	EAC03
Microsatellite 1.501	1	473,732	473,905	107	107	107
Microsatellite 3.502	3	451,079	451,266	142, 174	174	174
Microsatellite 3.27	3	493,033	493,402	271, 299	271	271, 259
*msp4*	4	627,608	628,049	197, 221	197	197
*msp5*	4	630,303	630,795	330, 360	360	360
*msp1F3*	7	1,161,359	1,161,802	259, 253	250	250
Microsatellite MS16	9	1,593,168	1,593,791	220,100	256	256
*msp3a*	10	1,220,104	1,220,928	465	474	474

### Analysis of SNVs: Determination of genetic relatedness

We next sought to determine the genetic relatedness between the recurrent parasite infections using the genotyped markers. Genotyping at the 55,399 loci showed that 4,434 (32.8%) of the 13,536 genotyped positions (5 or more reads) that were variant between the three infections produced different base calls in the two relapse samples (Supporting Dataset 1), contradicting the microsatellite analysis showing that the two relapses are virtually identical (Table 3). This result initially suggested that these two episodes might have come from different infection events. To investigate further, EAC02 and EAC03 were subsequently subjected to a pairwise comparison in which confidently genotyped markers were plotted as a function of chromosome position (Figure 3). Surprisingly, these data indicate that the parasites exhibited a highly non-random pattern in which regions of identity were organized into large blocks with a weighted average size of 715 kb and that the microsatellite markers were located by chance in regions that happened to lack variant SNVs. As a control, the two relapse samples were also compared in a pairwise manner to the Brazil I strain since this strain is also believed to be derived from a relapse infection. We observed no evidence that the relapse samples shared large contiguous sequences of DNA with this South American strain (Figure 3). We additionally calculated the haplotype block sizes from pairwise comparisons between EAC02 and Brazil I and EAC02 and Mauritania 1 (the strain most closely related by PCA to the East African samples investigated here). The haplotype block sizes for these comparisons were 5.8 kb and 6.3 kb, respectively, indicating that EAC02 shared no large contiguous pieces of DNA with these other *P. vivax* stains.

We next compared the two definitive relapse samples to the first sample (EAC01) at those loci that were unambiguously genotyped in all three infections. We again found that the strains obtained from our patient over 33 months shared substantial portions of the *P. vivax* genome and that the regions of identity were organized into contiguous blocks of genomic sequence (data not shown and Supporting Dataset 1). These analyses further suggest that parasites from all three infections are highly related, yet genetically distinct, to one another.

Since EAC01 was a polyclonal infection, we sought to determine if it was comprised exclusively of parasites directly related to EAC02 and EAC03. If EAC01 only contained parasites that were directly related to the second and third infections, then genotyped loci in EAC01 would not contain more than two alleles. Of the 10,775 loci that contained more than one allele in EAC01 only six loci (5.6×10^−4^%) possessed more than two alleles with high confidence (greater than 5 reads). These data suggest that the first infection is comprised of two or more parasites directly related to EAC02 and EAC03. Additionally, when EAC01 variant mixed-read alleles from chromosome one were sorted by read count (Table 4) one resulting haplotype perfectly matched that of EAC02 and EAC03 (which are identical on chromosome 1) and the other was completely different. These data suggest that either there are three clones, one with the chromosome 1 EAC02/03 haplotype and two with the alternative haplotype, or that there are only two different clones present, but that EAC02/03 haplotype clone is less abundant (Table 3).

**Table 4:** Read counts on chromosome 1 allow separation of haplotypes.

		EAC01				EAC02				EAC03			
		Major		Minor		Major		Minor		Major		Minor	
Chr	Pos.	Call	RC	Call	RC	Call	RC	Call	RC	Call	RC	Call	RC
1	13468	C	22	T	19	T	50	none	0	T	49	none	0
1	13786	G	37	A	12	A	48	none	0	A	48	none	0
1	19230	G	29	A	21	A	50	none	0	A	50	none	0
1	19298	G	29	C	21	C	50	none	0	C	49	none	0
1	28285	C	23	T	19	T	49	none	0	T	50	none	0
1	29187	C	10	T	6	T	44	none	0	T	50	none	0
1	29396	G	9	C	3	C	42	none	0	C	50	none	0
1	29421	C	11	T	4	T	40	none	0	T	49	none	0
1	31132	C	26	T	24	T	49	none	0	T	50	none	0
1	32817	T	32	A	17	A	49	none	0	A	50	none	0
1	35070	C	30	G	20	G	50	none	0	G	50	none	0
1	35824	C	28	T	21	T	50	none	0	T	49	none	0
1	36851	C	25	T	21	T	49	none	0	T	49	none	0
1	38321	G	30	T	20	T	50	none	0	T	50	none	0
1	42102	G	27	A	14	A	50	none	0	A	50	none	0
1	45320	T	23	G	19	G	50	none	0	G	50	none	0
1	46645	T	9	A	4	A	26	none	0	A	44	none	0
1	48694	G	35	A	15	A	50	none	0	A	50	none	0
1	49988	C	17	T	10	T	50	none	0	T	50	none	0

Data from the first 50 kb are shown. Only genotyped positions that are different between the three infections and which have 5 or more reads are shown. RC = read count.

Overall these data suggest that EAC02 and EAC03 and the two clones in EAC01 are separated by only a single meiosis. In the related human parasite, *P. falciparum*, sexual crosses performed using chimpanzees showed that the average number of bases per 100 recombination events (one centimorgan = 10 kb) is 9.6 kb [33]. Based on the 27 breakpoints shown in Figure 3 over the 26.9 Mb *P. vivax* genome [34] we estimate that the average number of bases per 100 recombinations is a similar 10 kb. Although no laboratory crosses of *P. vivax* have been performed, our clones appear to be the progeny of a natural cross.

### Reciprocal recombination events

**Figure 4:**
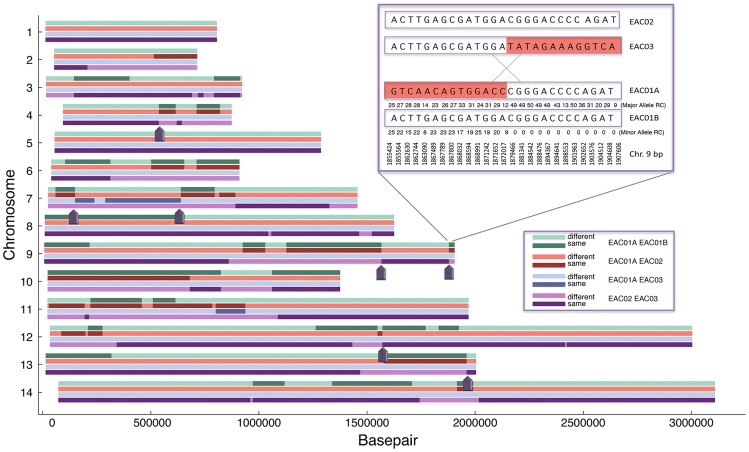
Multiple two-way comparisons show evidence of reciprocal recombination events. To ensure that only high quality positions would be used, the 55,399 genotyped loci were stringently filtered. The filtering criteria was that there had to be more than 20 read counts for the major allele of each comparison (EAC01, EAC02, and EAC03), and that all three strains' SNV positions differed from the reference. A likelihood function was generated examining 10,000 basepair windows for recombination and assumed that each transition (same to different in each pairwise comparison taken individually) could be considered a chance of recombination. A smoothing kernel was applied to differentiate if a position was likely to be the same by chance or due to recombination and depended on the distance of identical neighboring SNVs (the further separated identical SNVs were, the chances of a true recombination event was much less). Only edges with Z scores greater than 4.188 (99th percentile) were taken as true edges. For the inset, the numbers below the line represent read count. On the right side of the recombination event, there is no minor allele in EAC01 and thus EAC01A and EAC01B are presumed to be identical, while on the left side, the minor allele (EAC01B) read count is relatively high. RC = Read Count. Dark arrows indicate reciprocal recombination events found in three or more comparisons.

The malaria parasite undergoes sexual reproduction during the mosquito life cycle stage including recombination between male and female gametes. Since the infections were most likely caused by “sibling” parasites, we next sought to determine if they had arisen from a single zygote and if there was evidence of reciprocal recombination events, which would indicate a direct genetic relationship between the parasite strains from the recurrent infections. To accomplish this task we analyzed the whole genome sequencing data in multiple two way comparisons that separated the two clones in EAC01 into two different virtual clones (EAC01A and EAC01B) based on read count (Figure 4). While it is recognized that this is not ideal because stochastic differences in read count are highly likely, without the parental haplotype a third sample is, nevertheless, necessary to visualize reciprocal events. This is because although a recombination event can be found that occurred in one sibling but not the other using WGS, reciprocal events would be invisible. Because of the potential for noise we further filtered the set to include only the highest quality base calls. Specifically, we used a dataset of 5938 positions that had at least 20 reads across EAC01, EAC02, and EAC03 major alleles. Regions were plotted across the chromosome and colored based on whether they were identical or different. Comparisons between EAC01A and EAC01B, EAC02, and EAC03 and a fourth comparison between EAC02 and EAC03 are shown in Figure 4.

The two clones (EAC02 and EAC03) and the two virtual clones (EAC01A and EAC01B) were identical across much of the genome. As suggested previously, on chromosome 1, EAC01B, EAC02 and EAC03 were almost identical (different at 1 of 94 loci genotyped), but different from EAC01A at 93 of these loci. It is not clear that the microsatellite genotyping (Microsatellite 1.501, Table 3) would have detected the EAC01A virtual clone, but conventional microsatellite genotyping of chromosome 1 showed only 1 product in all three isolates for marker 1.501. Likewise, there was no evidence of recombination on chromosome 5 (183 of 183 loci identical in EAC01B, EAC02, and EAC03). Nevertheless, clear evidence of reciprocal recombination events were visible on all other chromosomes with most chromosomes having between 1 and 4. These breakpoints were identified as areas of the genome where one clone transitions from sharing a haplotype to not sharing a haplotype (Figure 4). It should be noted that breakpoints detected using read counts of the two virtual clones (EAC01A and EAC01B) were confirmed with the two independently sequenced samples, EAC02 and EAC03. An example of one of these events on chromosome 9 at base pair resolution is shown in Figure 4. The high-resolution data show that the recombination event occurred between bases 1,872,017 and 1,879,466. The four clones analyzed here share 21 recombination breakpoints, which is in concordance with the number of crossovers postulated to occur per meiosis in *P. falciparum* [33], [35].

### Regions of homozygosity and drug resistance

As noted above, the patient here was treated with primaquine after both the first and second infection, yet still suffered additional relapse infections, suggesting that the parasites could be resistant to primaquine. Although the number of samples here is too small to map a resistance gene we nevertheless looked to see if known drug resistance genes were heterozygous or homozygous across the different infections and thus could indicate evidence of positive selection.

The *P. vivax pfcrt* homolog (PVX_087980) on chromosome 1 was identical across all three infections with only two synonymous mutations (D328 and F339) in comparison to the SalI reference strain within a region of homozygosity that spanned 11,912 bases. For the multidrug resistance loci *pvmdr* (PVX_080100), *pvmrp* (PVX_097025), and *gtp cyclohydrolase* (PVX_123830) we found there were two haplotypes present across the three infections. But for *pvdhfr* (PVX_089950) and *pvdhps* (PVX_123230), genes involved in anti-folate resistance, there was only one haplotype across all three infections, revealing an area known to be under selection in that region of the world [36]. For *pvdhfr* the region of homozygosity on chromosome 5 was 23 kb, but for *pvdhps* on chromosome 14, the region of homozygosity spanned 132 kb.

Along with SNVs in putative resistance genes, we also looked for copy number variants (CNV), a key mechanism of resistance in *P. falciparum* [37]–[40], in *P. vivax* homologs of known *P. falciparum* drug resistance genes (Table 5). In the three infections analyzed here, we were unable to detect any amplification CNVs (>2.0 sequencing coverage relative to the genomic average) at the genes of interest using a novel CNV detection algorithm [22]. In addition, we did not detect any significant differences in *relative* sequencing coverage between the three infections. However, although the algorithm used here (see Methods) has been shown to robustly identify CNVs in *P. falciparum*, in the absence of genome-captured *P. vivax* DNA samples with known CNVs that could serve as positive controls, false negatives could be possible.

**Table 5:** Copy number variants in putative drug resistance genes from relapsing clones.

				Relative Gene Coverage		
Gene	Chromosome	Start	End	EAC01	EAC02	EAC03
*pvcrt*	1	330,260	334,540	1.0419	1.0451	1.0438
PVX_087980						
*pvmrp*	2	153,642	158,822	0.7052	0.7406	0.7355
PVX_097025						
*pvdhfr*	5	964,590	966,469	0.6123	0.6134	0.6233
PVX_089950						
*pvmdr1*	10	361,490	366,095	1.4668	1.4562	1.4432
PVX_080100						
*pvdhps*	14	1,256,701	1,259,581	1.1466	1.1476	1.1538
PVX_123230						
*pvgtp-cyclohydrolase*	14	1,824,997	1,826,268	0.7978	0.7781	0.7839
PVX_123830						

## Discussion

Here we show whole genome sequencing results for three sequential recurrent *P. vivax* infections, two of which could be definitively categorized as relapse infections, occurring in a patient over 33 months in a non-endemic country. These results demonstrate the feasibility of using the whole genome capture technique on archived samples after long term storage along with standard samples directly from the field to dramatically improve our understanding of *P. vivax* infections within a single patient.

These data also demonstrate the power available from the more comprehensive datasets produced with whole genome technologies to more accurately describe the genetic structure both within a geographic region and also temporally within a single patient. Comparing the whole genome sequencing data to the current standard of RFLP on 8–15 markers demonstrates that data from small genotyping sets are more susceptible to common errors. For instance EAC03 had three bands by RFLP analysis at the microsatellite 3.27, but analysis of this region by whole genome sequencing using hundreds of markers showed this region to be clonal. The most likely cause for this error is a PCR error, and while whole genome sequencing is not immune to PCR errors the high level of read counts at each individual locus along with the dense set of markers across the entire genome make the technique more robust than the current standard genotyping methods.

Additionally, as the cost of sequencing declines, new methods like whole genome capture will make it feasible to generate larger more complete genetic datasets of malaria infections. These new datasets will allow more robust characterization of recurrent infections; better classification of recurrent *P. vivax* infections will be crucial in designing next generation anti-malarials as well as public health interventions directed specifically at *P. vivax*.

The latter two of the three recurrent malaria infections analyzed here can be definitively classified as relapse infections, which arose from the activation of hypnozoites. Prior to each of these two infections the patient had been negative for malaria infection by diagnostic PCR, ruling out a recrudescence. Furthermore, the patient history stated that the patient had not been to a malaria endemic country since the previous malaria infection, ruling out a reinfection.

Both of the definitive relapse samples (EAC02 and EAC03) were clonal based on analysis of the whole genome sequencing data. This suggests that a single hypnozoite was activated through an unknown trigger and emerged from the liver to cause symptomatic disease. In contrast the first recurrent infection, whose origin could not be distinguished between a relapse and recrudescence, was polyclonal. These results are in concordance with previous studies looking at relapse samples from patients with limited or no previous malaria infection [5], [6]. Additionally, the clonality seen in the final infection might be a result of the extended course of primaquine the patient received after the second infection. This treatment regimen might have selected only one highly resistant hypnozoite leading to a clonal infection as an artifact of treatment. Therefore clonality might be suggested as a marker for relapse infection *only* in these specific circumstances. In contrast, reports from the literature indicate that relapse infections occurring in endemic areas can be either clonal or polyclonal due to the hypnozoite load in the liver that has accumulated from previous mosquito inoculations [8]–[10]. The high degree of relatedness between the two infections also corroborates the patient's medical history indicating that the parasites causing these two infections separated by 30 months were acquired in the same region at the same time. If the patient had travelled to another malaria endemic region, the parasites causing infection would be genetically different, and, if the patient had returned to East Africa, the recombination breakpoints would have been different. These data also support the idea that the parasites are from the same sub-geographic region within East Africa and not a combination of parasites from Sudan and Eritrea, for instance.

From a public health perspective, these data highlight the need for analyzing *P. vivax* samples from the field with a denser genetic array than has previously been performed. While EAC02 and EAC03 are highly related as described above, there are still substantial portions of the *P. vivax* genome where they differ. Nevertheless, the traditional eight-marker genotyping panel indicated that these two parasites were identical at all eight markers suggesting that these two infections were clones of one another, which is not corroborated by the denser genomic data. Also, looking at the pairwise comparison between EAC02 and EAC03 demonstrates that selecting a different set of eight random markers from the genome could just as easily have indicated the parasites where completely unrelated. We demonstrate here that the current gold standard techniques investigating 8–15 genes or microsatellites will be unable to fully describe the complex genetic structure of *P. vivax* infections.

**Figure 5:**
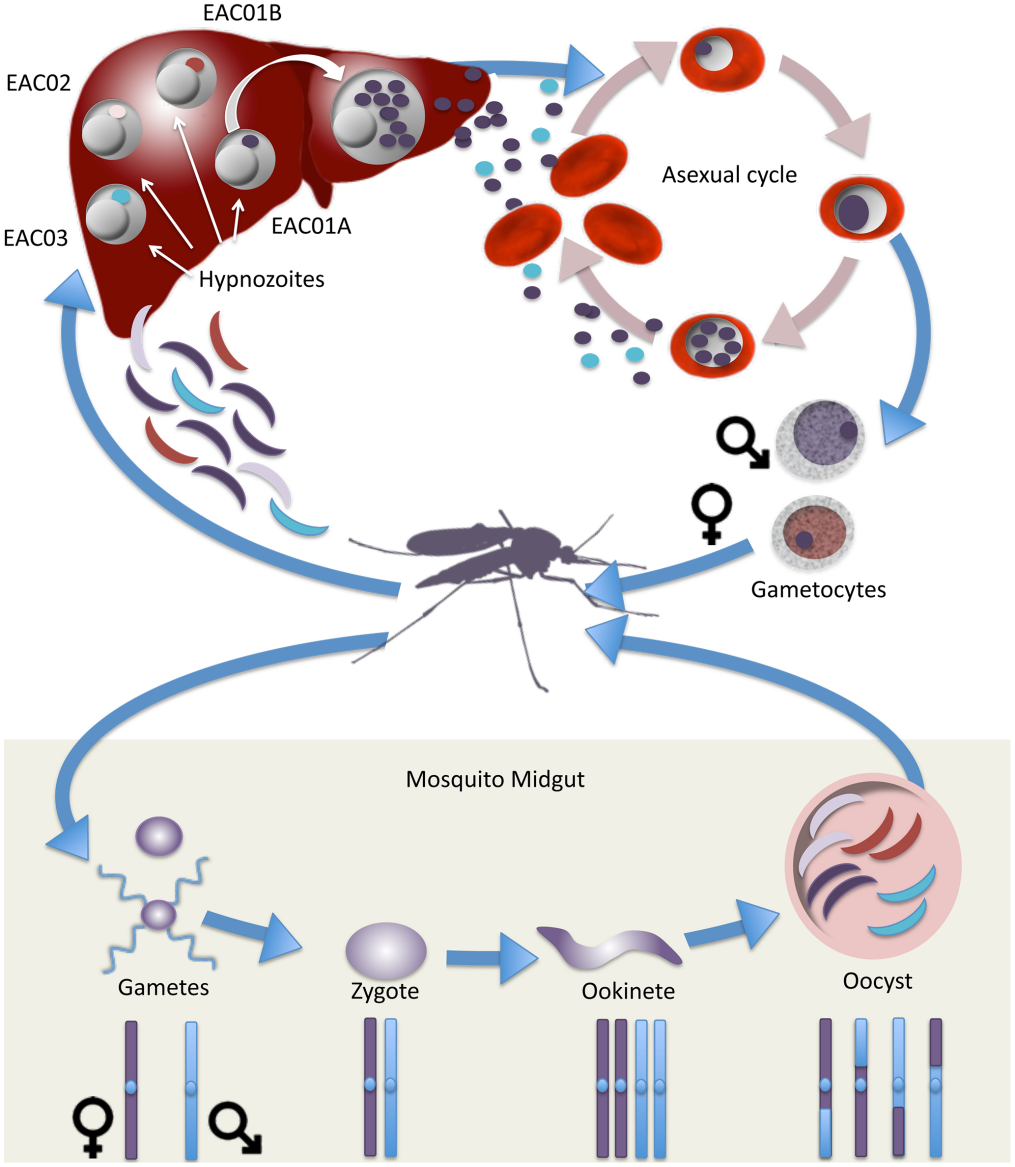
Genetic cycle of *P. vivax* relapse predicted from whole genome sequencing. The primary infection (EAC01) is polyclonal with at least two, and possibly more, meiotic siblings. It is inferred that this infection came from the activation of two different hypnozoites (EAC01A and EAC01B). Based on the higher number of reads from EAC01A, this hypnozoite may have been activated first. It is also possible that asexual parasites descended from a third meiotic sibling are present in the EAC01 infection but its DNA was poorly amplified. The two relapses (EAC02 and EAC03) are predicted to be clonal. This model is based on relapses coming from a single hypnozoite, which may be rare in regions where individuals are repeatedly infected with *P. vivax* and where there may be many circulating haplotypes.

Further genomic analysis of the two definitive relapse samples indicates that these two infections are highly related. These two infections share large amounts of DNA, and the DNA that is homologous between these two *P. vivax* strains is organized into large contiguous blocks of genomic DNA, or haplotypes. The average size of the haplotype blocks shared by these two parasite strains (715 kb) is roughly half the size of the average chromosome (1.5 Mb) indicating that these two samples are separated by a single round of meiosis (Figure 5). Comparing the second and third infection to the first infection also indicated that these parasite strains arose from the same parental gametes. Additionally, genomic analysis demonstrated that these three parasite samples shared reciprocal recombination breakpoints. While it is not uncommon to find distinct recombination breakpoints in malaria samples collected from a specific region, especially one where transmission is quite low [41], [42], the fact that reciprocal recombination breakpoints were found, and that all recombination breakpoints identified were reciprocal, further illustrates the direct genetic relatedness of these three infections.

In single-celled eukaryotes such as *S. cerevisiae*, a single zygote can give rise to four meiotic progeny that constitute a “tetrad” and show reciprocal recombination breakpoints. We hypothesize based on the recombination data that the direct genetic relationship hypothesized here is of meiotic siblings from the same zygote. This meiotic sibling hypothesis would imply that sporozoites from a single viable oocyst were injected into the patient at the time of the infectious mosquito bite. Due to the low transmission rate in the area of infection and the low oocyst burden of mosquitoes seen in the field, this is the most likely scenario.

A potential alternative to the meiotic sibling hypothesis is that the parasites seen here constitute a family trio. This parent-child relationship could be seen one of two ways. One, the patient might have been infected by three separate mosquitoes each carrying a separate member (mother, father, and child) of the trio. Further analysis of the epidemiology of *P. vivax* in East Africa and, in particular in Sudan, indicates that a parent-child relationship between multiple infections, while genetically feasible, is unlikely due to low transmission rates in the area [41], [42].

The second mechanism by which the patient could have been infected by this nuclear family of parasites is for the patient to have been infected by one mosquito harboring multiple oocysts. In the parent-child relationship structure, sporozoites from at least three oocysts would have had to be injected into the patient: one with the cross, and two self-crosses for each of the parental strains. In addition, there would have most likely been additional oocysts present that contained additional crosses that would have also been injected. Recombination data to date from the initial polyclonal infection has not found evidence of additional parasite crosses between two putative parental strains further suggesting that the genetic relationship between these parasites are not a parent-child relationship.

There is also the possibility that there was previously one cross in the region of inoculation with the four descendants of this cross circulating in the area and maintaining themselves by consecutive self-crosses in the mosquito. Under this hypothesis, the patient in this study would have been sequentially infected by three of these strains. Based on the data and malaria transmission characteristics in the Sudan, we believe this hypothesis is of low probability for two reasons. One, the patient is unlikely to have received three infectious bites during his short stay in Sudan, an area of low malaria transmission. Two, if the sexual cross had occurred more than one generation ago, it is unlikely that the parasites would share all recombination breakpoints. For this to happen, the parasites from the initial cross would have to have been segregated via multiple human infections or multiple mosquitoes immediately after the initial cross since otherwise the chance of all four progeny only self-crossing in the next generation is unlikely. In this area of low transmission it is unlikely that multiple mosquitoes would have transmitted progeny from the initial cross to three or four other individuals and it is equally unlikely that the same mosquito would have infected three of four other people, each with a different progeny from the same cross. For these two reasons, both relating to the low transmission dynamics of the region of inoculation, the possibility of the cross seen here occurring >1 generation ago is small.

We of course cannot definitively distinguish between the alternative hypothesis relationships without the putative fourth member of the tetrad. Despite this fact, the meiotic sibling hypothesis is the most likely of the proposed hypotheses at this time as it best conforms to the *P. vivax* population structure and transmission rate present in Sudan.

Additionally, the dense genetic maps obtained via whole genome sequencing highlight a new, unique aspect of *P. vivax* immune evasion. It is known from therapeutic malaria experiments in the first half of the 20^th^ century that patients can become immune to successive relapse infections [3]. However, these infections were initiated by only a few strains usually propagated by blood transfusion which were likely genetically identical. Here we suggest that in a natural infection resulting from the sexual cross of two different parasites, up to four genetically unique parasites emerge from each oocyst (Figure 5). These diverse parasites have a greater chance to evade the human immune response as they are subsequently activated and emerge from the liver. These high-resolution data in which haplotypes can be distinguished simply based on read count offer the opportunity to use linkage analysis of complex patient samples in mapping drug resistance genes. Higher levels of read coverage could also allow physical mapping and *de novo* assembly of clones in a mixed infection.

Whole genome sequencing of sequential *P. vivax* infections including definitive relapses also strengthens a classic model of *P. vivax* biology with genomic data. Studies in the first half of the 20^th^ century of *P. vivax* relapse using malaria therapy for neurosyphilis as a model demonstrated that both a short latency relapse and a long latency relapse can arise from parasites that are directly related to one another [43]. Confirming these initial results, we show here on the genomic level that genetically related parasites from the same cross can cause both a short and long latency relapse infection. This further confirms that *P. vivax* parasites are inherently capable of remaining dormant in the liver for months to years unless activated by the appropriate (unknown) trigger. These data also highlight the need to better understand the trigger that activates hypnozoites, currently hypothesized to be a febrile illness [44], and/or hypnozoite biomarkers that can be used for long-term surveillance for *P. vivax* infections in tropical areas as part of malaria elimination programs.

Another area of interest highlighted by this particular case is primaquine resistance. The patient was treated with a standard dose of primaquine after the initial infection and a double dose after the first relapse, but the parasites were obviously able to evade this treatment. At this point, it is unclear whether the failure of primaquine was due to parasite resistance or the patient's inability to metabolize primaquine to its active form. If these genetically related parasites are resistant to primaquine then it is possible that the patient initially harbored a more diverse hypnozoite load in the liver that was cleared after primaquine treatment. However, analysis of the initial recurrent infection, obtained before primaquine treatment, does not suggest this to be the case, but it cannot be definitively ruled out. This patient's history nevertheless demonstrates that primaquine is not always effective and that safe, efficacious replacements are needed. A major obstacle to both testing new anti-hypnozoite drugs and for monitoring the effectiveness of primaquine in endemic countries is the inability to distinguish between the sources of recurrent infection.

This study is limited by its inherent uniqueness. With only three samples to date, we are unable to definitively answer many of the outstanding questions, such as the exact genetic relationship between strains and primaquine resistance. In addition, the answer to the relationship structure between the three infections would be more easily answered if the *P. vivax* community possessed a more solid understanding of the population structure in Sudan, but due to the political instability in the region this is currently not feasible. In order to overcome these limitations, more relapse samples, and preferably multiple sequential relapse samples from a single patient, need to be obtained. These infections can be either naturally occurring or controlled *P. vivax* infections based on new protocols [45].

Further in depth analysis of definitive relapse infections will shed more light on this crucial parasite stage, but, as demonstrated in this study, current relapse analysis methods lack the power to fully characterize the hypnozoite stage for either research or public health purposes. The hypnozoite will therefore need to be the focus of specific intervention programs if the goal of malaria elimination is to be realized in areas endemic for *P. vivax*.

## Supporting Information

Dataset S1 Genotyping data from the three samples sequenced in this study (EAC01-03) and five publicly available *P. vivax* samples for the genomic loci in the genotyping set (see Methods). SNVs in genes are noted along with whether they are synonymous or non-synonymous base pair changes.
(XLSX)

## References

[1] Guerra CA, Howes RE, Patil AP, Gething PW, Van Boeckel TP, et al (2010) The international limits and population at risk of Plasmodium vivax transmission in 2009. PLoS Negl Trop Dis 4: e774 10.1371/journal.pntd.0000774 20689816 PMC2914753

[2] Baird JK (2009) Resistance to therapies for infection by Plasmodium vivax. Clin Microbiol Rev 22: 508–534 10.1128/CMR.00008-09 19597012 PMC2708388

[3] White NJ (2011) Determinants of relapse periodicity in Plasmodium vivax malaria. Malar J 10: 297 10.1186/1475-2875-10-297 21989376 PMC3228849

[4] White NJ, Imwong M (2012) Relapse. Adv Parasitol 80: 113–150 10.1016/B978-0-12-397900-1.00002-5 23199487

[5] Imwong M, Boel ME, Pagornrat W, Pimanpanarak M, McGready R, et al (2012) The first Plasmodium vivax relapses of life are usually genetically homologous. J Infect Dis 205: 680–683 10.1093/infdis/jir806 22194628 PMC3266132

[6] Chen N, Auliff A, Rieckmann K, Gatton M, Cheng Q (2007) Relapses of Plasmodium vivax infection result from clonal hypnozoites activated at predetermined intervals. J Infect Dis 195: 934–941 10.1086/512242 17330782

[7] Abdullah NR, Barber BE, William T, Norahmad NA, Satsu UR, et al (2013) Plasmodium vivax Population Structure and Transmission Dynamics in Sabah Malaysia. PLoS One 8: e82553 10.1371/journal.pone.0082553 24358203 PMC3866266

[8] Imwong M, Snounou G, Pukrittayakamee S, Tanomsing N, Kim JR, et al (2007) Relapses of Plasmodium vivax infection usually result from activation of heterologous hypnozoites. J Infect Dis 195: 927–933 10.1086/512241 17330781

[9] Kim J-R, Nandy A, Maji AK, Addy M, Dondorp AM, et al (2012) Genotyping of Plasmodium vivax Reveals Both Short and Long Latency Relapse Patterns in Kolkata. PLoS One 7: e39645 10.1371/journal.pone.0039645 22808048 PMC3396609

[10] De Araujo FCF, de Rezende AM, Fontes CJF, Carvalho LH, Alves de Brito CF (2012) Multiple-clone activation of hypnozoites is the leading cause of relapse in Plasmodium vivax infection. PLoS One 7: e49871 10.1371/journal.pone.0049871 23185469 PMC3503861

[11] Thanapongpichat S, McGready R, Luxemburger C, Day NPJ, White NJ, et al (2013) Microsatellite genotyping of Plasmodium vivax infections and their relapses in pregnant and non-pregnant patients on the Thai-Myanmar border. Malar J 12: 275 10.1186/1475-2875-12-275 23915022 PMC3750759

[12] Bright AT, Alenazi T, Shokoples S, Tarning J, Paganotti GM, et al (2013) Genetic analysis of primaquine tolerance in a patient with relapsing vivax malaria. Emerg Infect Dis 19: 802–805 10.3201/eid1905.121852 23648098 PMC3647516

[13] Koepfli C, Mueller I, Marfurt J, Goroti M, Sie A, et al (2009) Evaluation of Plasmodium vivax genotyping markers for molecular monitoring in clinical trials. J Infect Dis 199: 1074–1080 10.1086/597303 19275476

[14] Bright AT, Tewhey R, Abeles S, Chuquiyauri R, Llanos-Cuentas A, et al (2012) Whole genome sequencing analysis of Plasmodium vivax using whole genome capture. BMC Genomics 13: 262 10.1186/1471-2164-13-262 22721170 PMC3410760

[15] Tewhey R, Nakano M, Wang X, Pabón-Peña C, Novak B, et al (2009) Enrichment of sequencing targets from the human genome by solution hybridization. Genome Biol 10: R116 10.1186/gb-2009-10-10-r116 19835619 PMC2784331

[16] Li H, Durbin R (2009) Fast and accurate short read alignment with Burrows-Wheeler transform. Bioinformatics 25: 1754–1760 10.1093/bioinformatics/btp324 19451168 PMC2705234

[17] DePristo MA, Banks E, Poplin R, Garimella K V, Maguire JR, et al (2011) A framework for variation discovery and genotyping using next-generation DNA sequencing data. Nat Genet 43: 491–498 10.1038/ng.806 21478889 PMC3083463

[18] McKenna A, Hanna M, Banks E, Sivachenko A, Cibulskis K, et al (2010) The Genome Analysis Toolkit: a MapReduce framework for analyzing next-generation DNA sequencing data. Genome Res 20: 1297–1303 10.1101/gr.107524.110 20644199 PMC2928508

[19] Li H, Handsaker B, Wysoker A, Fennell T, Ruan J, et al (2009) The Sequence Alignment/Map format and SAMtools. Bioinformatics 25: 2078–2079 10.1093/bioinformatics/btp352 19505943 PMC2723002

[20] Cingolani P, Platts A, Wang LL, Coon M, Nguyen T, et al (2012) A program for annotating and predicting the effects of single nucleotide polymorphisms, SnpEff: SNPs in the genome of Drosophila melanogaster strain w1118; iso-2; iso-3. Fly (Austin) 6: 80–92 10.4161/fly.19695 22728672 PMC3679285

[21] Manske M, Miotto O, Campino S, Auburn S, Almagro-Garcia J, et al (2012) Analysis of Plasmodium falciparum diversity in natural infections by deep sequencing. Nature 10.1038/nature11174 PMC3738909 22722859

[22] Manary MJ, Singhakul SS, Flannery EL, Bopp SE, Corey VC, et al (2014) Identification of pathogen genomic variants through an integrated pipeline. BMC Bioinformatics 15: 63 10.1186/1471-2105-15-63 24589256 PMC3945619

[23] Dharia N V, Bright AT, Westenberger SJ, Barnes SW, Batalov S, et al (2010) Whole-genome sequencing and microarray analysis of ex vivo Plasmodium vivax reveal selective pressure on putative drug resistance genes. Proc Natl Acad Sci U S A 107: 20045–20050 10.1073/pnas.1003776107 21037109 PMC2993397

[24] Sriprawat K, Kaewpongsri S, Suwanarusk R, Leimanis ML, Lek-Uthai U, et al (2009) Effective and cheap removal of leukocytes and platelets from Plasmodium vivax infected blood. Malar J 8: 115 10.1186/1475-2875-8-115 19490618 PMC2694833

[25] Gnirke A, Melnikov A, Maguire J, Rogov P, LeProust EM, et al (2009) Solution hybrid selection with ultra-long oligonucleotides for massively parallel targeted sequencing. Nat Biotechnol 27: 182–189 10.1038/nbt.1523 19182786 PMC2663421

[26] Neafsey DE, Galinsky K, Jiang RH, Young L, Sykes SM, et al (2012) The malaria parasite Plasmodium vivax exhibits greater genetic diversity than Plasmodium falciparum. Nat Genet 44: 1046–1050 10.1038/ng.2373 22863733 PMC3432710

[27] Patterson N, Price AL, Reich D (2006) Population structure and eigenanalysis. PLoS Genet 2: e190 10.1371/journal.pgen.0020190 17194218 PMC1713260

[28] Auburn S, Campino S, Miotto O, Djimde A a, Zongo I, et al (2012) Characterization of within-host Plasmodium falciparum diversity using next-generation sequence data. PLoS One 7: e32891 10.1371/journal.pone.0032891 22393456 PMC3290604

[29] Craig AA, Kain KC (1996) Molecular analysis of strains of Plasmodium vivax from paired primary and relapse infections. J Infect Dis 174: 373–379. 8699069 10.1093/infdis/174.2.373

[30] Bopp SER, Manary MJ, Bright AT, Johnston GL, Dharia N V, et al (2013) Mitotic Evolution of Plasmodium falciparum Shows a Stable Core Genome but Recombination in Antigen Families. PLoS Genet 9: e1003293 10.1371/journal.pgen.1003293 23408914 PMC3567157

[31] Rayner JC, Huber CS, Feldman D, Ingravallo P, Galinski MR, et al (2004) Plasmodium vivax merozoite surface protein PvMSP-3 beta is radically polymorphic through mutation and large insertions and deletions. Infect Genet Evol 4: 309–319 10.1016/j.meegid.2004.03.003 15374528

[32] Galinski MR, Corredor-Medina C, Povoa M, Crosby J, Ingravallo P, et al (1999) Plasmodium vivax merozoite surface protein-3 contains coiled-coil motifs in an alanine-rich central domain. Mol Biochem Parasitol 101: 131–147. 10413049 10.1016/s0166-6851(99)00063-8

[33] Jiang H, Li N, Gopalan V, Zilversmit MM, Varma S, et al (2011) High recombination rates and hotspots in a Plasmodium falciparum genetic cross. Genome Biol 12: R33 10.1186/gb-2011-12-4-r33 21463505 PMC3218859

[34] Carlton J (2003) The Plasmodium vivax genome sequencing project. Trends Parasitol 19: 227–231. 12763429 10.1016/s1471-4922(03)00066-7

[35] Su X, Ferdig MT, Huang Y, Huynh CQ, Liu A, et al (1999) A genetic map and recombination parameters of the human malaria parasite Plasmodium falciparum. Science 286: 1351–1353 10.1126/science.286.5443.1351 10558988

[36] Mula P, Fernandez-Martinez A, de Lucio A, Ramos JM, Reyes F, et al (2011) Detection of high levels of mutations involved in anti-malarial drug resistance in Plasmodium falciparum and Plasmodium vivax at a rural hospital in southern Ethiopia. Malar J 10: 214 10.1186/1475-2875-10-214 21810256 PMC3161020

[37] Wilson CM, Serrano AE, Wasley A, Bogenschutz MP, Shankar AH, et al (1989) Amplification of a gene related to mammalian mdr genes in drug-resistant Plasmodium falciparum. Science 244: 1184–1186. 2658061 10.1126/science.2658061

[38] Cowman AF, Galatis D, Thompson JK (1994) Selection for mefloquine resistance in Plasmodium falciparum is linked to amplification of the pfmdr1 gene and cross-resistance to halofantrine and quinine. Proc Natl Acad Sci U S A 91: 1143–1147. 8302844 10.1073/pnas.91.3.1143 PMC521470

[39] Price RN, Uhlemann A-C, Brockman A, McGready R, Ashley E, et al (2004) Mefloquine resistance in Plasmodium falciparum and increased pfmdr1 gene copy number. Lancet 364: 438–447 10.1016/S0140-6736(04)16767-6 15288742 PMC4337987

[40] Kidgell C, Volkman SK, Daily J, Borevitz JO, Plouffe D, et al (2006) A systematic map of genetic variation in Plasmodium falciparum. PLoS Pathog 2: e57 10.1371/journal.ppat.0020057 16789840 PMC1480597

[41] Abdallah TM, Abdeen MT, Ahmed IS, Hamdan HZ, Magzoub M, et al (2013) Severe Plasmodium falciparum and Plasmodium vivax malaria among adults at Kassala Hospital, eastern Sudan. Malar J 12: 148 10.1186/1475-2875-12-148 23634728 PMC3655045

[42] Abdallah TM, Ali AA, Bakri M, Gasim GI, Musa IR, et al (2012) Efficacy of artemether-lumefantrine as a treatment for uncomplicated Plasmodium vivax malaria in eastern Sudan. Malar J 11: 404 10.1186/1475-2875-11-404 23217037 PMC3519545

[43] White NJ, Imwong M (2012) Relapse. Adv Parasitol 80: 113–150 10.1016/B978-0-12-397900-1.00002-5 23199487

[44] Shanks GD, White NJ (2013) The activation of vivax malaria hypnozoites by infectious diseases. Lancet Infect Dis 13: 900–906 10.1016/S1473-3099(13)70095-1 23809889

[45] McCarthy JS, Griffin PM, Sekuloski S, Bright a T, Rockett R, et al (2013) Experimentally Induced Blood-Stage Plasmodium vivax Infection in Healthy Volunteers. J Infect Dis 208: 1688–1694 10.1093/infdis/jit394 23908484 PMC3888148

